# Diversity of Transcriptional Regulatory Adaptation in *E. coli*

**DOI:** 10.1093/molbev/msae240

**Published:** 2024-11-12

**Authors:** Christopher Dalldorf, Ying Hefner, Richard Szubin, Josefin Johnsen, Elsayed Mohamed, Gaoyuan Li, Jayanth Krishnan, Adam M Feist, Bernhard O Palsson, Daniel C Zielinski

**Affiliations:** Department of Bioengineering, University of California, San Diego, La Jolla, CA, USA; Department of Bioengineering, University of California, San Diego, La Jolla, CA, USA; Department of Bioengineering, University of California, San Diego, La Jolla, CA, USA; Novo Nordisk Foundation Center for Biosustainability, Technical University of Denmark, 2800 Kongens, Lyngby, Denmark; Novo Nordisk Foundation Center for Biosustainability, Technical University of Denmark, 2800 Kongens, Lyngby, Denmark; Department of Bioengineering, University of California, San Diego, La Jolla, CA, USA; Department of Bioengineering, University of California, San Diego, La Jolla, CA, USA; Department of Bioengineering, University of California, San Diego, La Jolla, CA, USA; Novo Nordisk Foundation Center for Biosustainability, Technical University of Denmark, 2800 Kongens, Lyngby, Denmark; Department of Bioengineering, University of California, San Diego, La Jolla, CA, USA; Novo Nordisk Foundation Center for Biosustainability, Technical University of Denmark, 2800 Kongens, Lyngby, Denmark; Bioinformatics and Systems Biology Program, University of California, San Diego, La Jolla, CA, USA; Department of Pediatrics, University of California, San Diego, La Jolla, CA, USA; Center for Microbiome Innovation, University of California, San Diego, La Jolla, CA 92093, USA; Department of Bioengineering, University of California, San Diego, La Jolla, CA, USA

## Abstract

The transcriptional regulatory network (TRN) in bacteria is thought to rapidly evolve in response to selection pressures, modulating transcription factor (TF) activities and interactions. In order to probe the limits and mechanisms surrounding the short-term adaptability of the TRN, we generated, evolved, and characterized knockout (KO) strains in *Escherichia coli* for 11 regulators selected based on measured growth impact on glucose minimal media. All but one knockout strain (Δ*lrp*) were able to recover growth and did so requiring few convergent mutations. We found that the TF knockout adaptations could be divided into four categories: (i) Strains (Δ*argR,* Δ*basR,* Δ*lon,* Δ*zntR,* and Δ*zur*) that recovered growth without any regulator-specific adaptations, likely due to minimal activity of the regulator on the growth condition, (ii) Strains (Δ*cytR,* Δ*mlrA,* and Δ*ybaO*) that recovered growth without TF-specific mutations but with differential expression of regulators with overlapping regulons to the KO’ed TF, (iii) Strains (Δ*crp* and Δ*fur*) that recovered growth using convergent mutations within their regulatory networks, including regulated promoters and connected regulators, and (iv) Strains (Δ*lrp*) that were unable to fully recover growth, seemingly due to the broad connectivity of the TF within the TRN. Analyzing growth capabilities in evolved and unevolved strains indicated that growth adaptation can restore fitness to diverse substrates often despite a lack of TF-specific mutations. This work reveals the breadth of TRN adaptive mechanisms and suggests these mechanisms can be anticipated based on the network and functional context of the perturbed TFs.

## Introduction

The bacterial transcriptional regulatory network (TRN) adapts to environmental changes (Balleza et al. 2009) primarily through the action of transcription factors (TFs) (Madan Babu and Teichmann 2003). Research into TFs has revealed insights into their regulatory targets (Gao et al. 2021), mechanisms of action (Rigali et al. 2004), and roles in environmental responses (Balleza et al. 2009). Genetic mutation enables flexibility of TF activities and interactions both on long (Balleza et al. 2009) and short (<1,000 generation) timescales (Sandberg et al. 2019). TF-related mutations have been observed in laboratory evolution experiments (Anand et al. 2021, 2020; Pal et al. 2022), indicating that microbes can meet short-term environmental challenges through genetic modulation of the TRN. An investigation of how the TRN adapts in the short term to recover growth following strong perturbations would provide insights into both the mechanistic basis and limits of the plasticity of the TRN.

TFs have rich and complex evolutionary histories. TF families share high sequence similarity across species but the large differences in their regulons across species suggests rapid evolution of regulatory networks (Price et al. 2007). TFs have previously been shown to be mutational targets in both short- and long-term evolution experiments (Zinser and Kolter 2000; Crozat et al. 2011; Zion et al. 2024) and regulatory mutations are common in stationary phase experiments (Chib et al. 2017). Regulatory mutations have also been found in antibiotic-resistant *Escherichia coli* strains, including the selective modification of the expression of a multidrug efflux pump (Praski Alzrigat et al. 2017), and have been shown to drastically adjust and rewire metabolic flux (Long et al. 2018). Regulatory mutations are thus a valuable evolutionary mechanism to adjust to a diverse set of environmental changes.

Knockout adaptive laboratory evolution (KO-ALE) has previously been used to study evolutionary adjustments to genetic perturbations in *E. coli*. Metabolic gene KO-ALEs revealed multiple optimal phenotypes exist to alleviate bottlenecks created by the KO, all of which substantially recover growth (Charusanti et al. 2010; McCloskey et al. 2018). Certain TF KO-ALE experiments have been previously carried out, including a *crp* KO-ALE that found convergent mutations to *ptsG*, an important gene in the glucose phosphotransferase system (Pal et al. 2022), and a *pdhR* KO-ALE that resulted in targeted mutations to the Shine-Dalgarno sequences of genes in *pdhR's* regulon (Anand et al. 2021). Mutations to regulators themselves have also been found when the TRN is perturbed in other ways, such as an enrichment of *crp* mutations following the KO of adenylate cyclase that produces the *crp* effector cAMP (Sekowska et al. 2016) and convergent mutations to *oxyR* following evolution with oxidative stress (Anand et al. 2020). In addition to these specific KO-ALE studies, another grew the Keio collection on M9 minimal media supplemented with glucose and found growth defects in KOs of TFs with no known function under this condition (Campos et al. 2018).

**Fig. 1.**
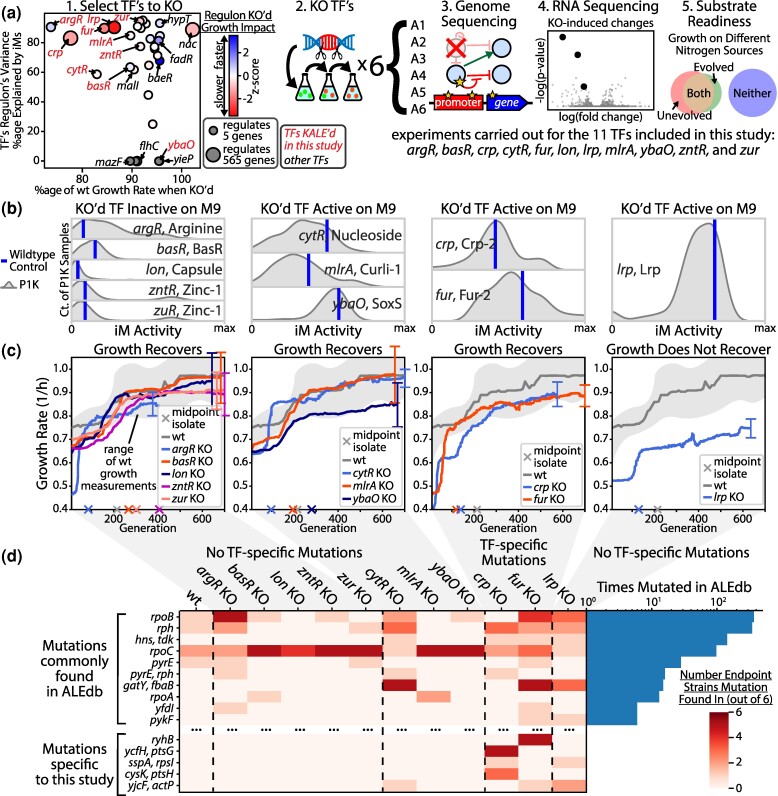
Experimental workflow and how KO strains group based on baseline regulator activity and recovery strategy. a) Eleven regulators were selected for KO-ALE whose labels are colored red in the left panel. With the exception of nac, only regulators with a negative impact to growth when removed are shown (Campos et al. 2018). The coloring for the leftmost figure is set for each regulator based on the combined impact to growth rate of all genes repressed by the regulator subtracted by the combined impact of all genes promoted by the regulator. The presented z-scores are from this distribution of totaled scores. The resulting evolved strains were sequenced and expression profiles were obtained for a select subset. Substrate readiness phenotyping was carried out. b) The activity level of the iModulons regulated by the regulator KO's. The gene and an iModulon it regulates are named in each subplot (gene, iModulon). These iModulon activities are corrected for basal activity, where zero represents no iModulon activity and all activity values are positive. See Methods: Basal iModulon Analysis for details. The gray curve is the distribution of PRECISE1K samples drawn using a kernel density estimation. c) Growth profiles for each of the KO's. Growth measurements were taken for each of the 6 independent lineages and the profiles shown are calculated based on a moving average of the growth measurements from all independent lineages. Said independent lineages are visible in supplementary fig. S1, Supplementary Material online. The vertical bars are 95% confidence intervals on the growth curves for each KO. d) A mutation table showing the genes mutated across the strains in this study. Some mutations have been removed from this figure for visual simplicity. The right histogram shows how many instances of each mutation can be found in ALEdb (including this study).

To obtain a more comprehensive understanding of short-term TRN plasticity, we performed the largest to-date study of the adaptive response to the removal of regulatory proteins in *E. coli*. We carried out KO-ALEs for 11 regulators, each with 6 independent lineages. The resulting midpoint and endpoint strains were sequenced, expression profiled, and characterized for substrate readiness using phenotyping plates. Independent component analysis (ICA) was used to analyze the gene expression response of the resulting strains, empowering the analysis through comparison to a broad range of experimental conditions in the PRECISE 1 K *E. coli* gene expression database (Sastry et al. 2019; Lamoureux et al. 2023) (Fig. 1a). The results of these experiments suggest a mutation landscape and network structure that is capable of rapidly responding to large perturbations and reveal the breadth of mechanisms underlying the adaptability of the *E. coli* TRN.

## Results

### Selection of Regulators for KO and Laboratory Evolution

In selecting regulators for KO-ALE, we sought deletions that would have a functional impact and therefore elicit targeted adaptations. We also prioritized regulators that have well characterized regulons to enable the estimation of condition-specific regulator activity. To estimate regulator activity, we utilized data-derived transcription modules called iModulons, which are machine learning-computed transcription regulatory modules that have been extensively analyzed on thousands of experimental conditions (Lamoureux et al. 2023). Regulators were therefore selected for KO-ALE based on three primary criteria (visualized in Fig. 1a): the regulon size based on binding sites annotated in regulonDB (Tierrafría et al. 2022), the growth rate impact of the KO on glucose M9 minimal media (Campos et al. 2018), and how well the regulator-associated iModulon explains the variance in expression of genes in the regulator's regulon.

These criteria resulted in 13 targets, 11 of which showed at least a 20% initial growth defect and were subsequently selected to move forward with ALE. Campos et al. (2018) measured these growth rates using 96-well plates while our strains were grown in flasks, perhaps explaining part of the discrepancy between our results and theirs. Each of these 11 KO strains were evolved in 6 separate lineages with midpoint and endpoint evolution isolates taken from each lineage. All isolates were sequenced to identify genetic mutations, the results of which informed the selection of a subset of the isolates to be expression profiled (see supplementary table S1, Supplementary Material online for details). *Lon*, a protease and not a TF, has been shown to selectively degrade certain proteins and thus have a regulatory role, which led to its inclusion in this study (Kirthika et al. 2023).

### Regulatory Activity on the Growth Condition Differs Between Deleted Transcription Factors

To anticipate the degree of impact of each regulator KO, we classified regulators based on the degree of regulatory activity on glucose minimal media (Fig. 1b). Each iM activity was oriented (in a mathematically equivalent transformation) to have approximately 0 as a minimum value, such that a larger activity value can be interpreted as a greater degree of regulatory activity (see Methods: Basal iModulon Transformation). Based on the activity levels of our wildtype control samples and the genetic adaptations seen in the evolved strains, we classify the regulator KO strains into four primary categories that can be seen in Fig. 1b–d: (i) regulator is inactive on M9 and growth recovers through nonregulator specific mutations (*argR, basR, lon, zntR,* and *zur*), (ii) regulator is active on M9 and growth recovers through nonregulator specific mutations (*cytR, mlrA,* and *ybaO*), (iii) regulator is active on M9 and growth recovers through regulator specific mutations (*crp* and *fur*), and (iv) regulator is active on M9 and there is poor growth recovery with nonregulator specific mutations (*lrp*). We discuss each of these categories below. Unless otherwise specified, all other figures and analyses outside of this classification step use the standard PRECISE1K (P1K) (Lamoureux et al. 2023) version of iModulons publicly available at iModulonDB.org.

### Almost all Regulator Knockout Strains Can Recover Growth With Only Few Mutations

The growth rates seen in Fig. 1c show that all of the KOs, except for *lrp,* nearly fully recover their growth rates. The *ybaO* KO strains were considered to have recovered growth as their growth data overlaps with the wildtype growth measurements which *lrp*'s growth data does not (see supplementary fig. S1, Supplementary Material online). All of the TF KO-ALE strains except for *crp* and *fur* did not exhibit any TF-specific causal mutations throughout their evolution (Fig. 1d). For these strains, mutations were largely limited to non-convergent RNA polymerase (RNAP) mutations (see supplementary File S1, Supplementary Material online) and expression differences primarily consisted of the downregulation of stress-related genes contained in the RpoS iModulon and the upregulation of ribosomal subunits contained in the Translation iModulon (supplementary fig. S2, Supplementary Material online). These adaptations are common across laboratory evolution experiments (Dalldorf et al. 2023).

### Adaptation to Removal of Active Regulators With Small Regulons Occurs Through Compensatory Mechanisms

**Fig. 2.**
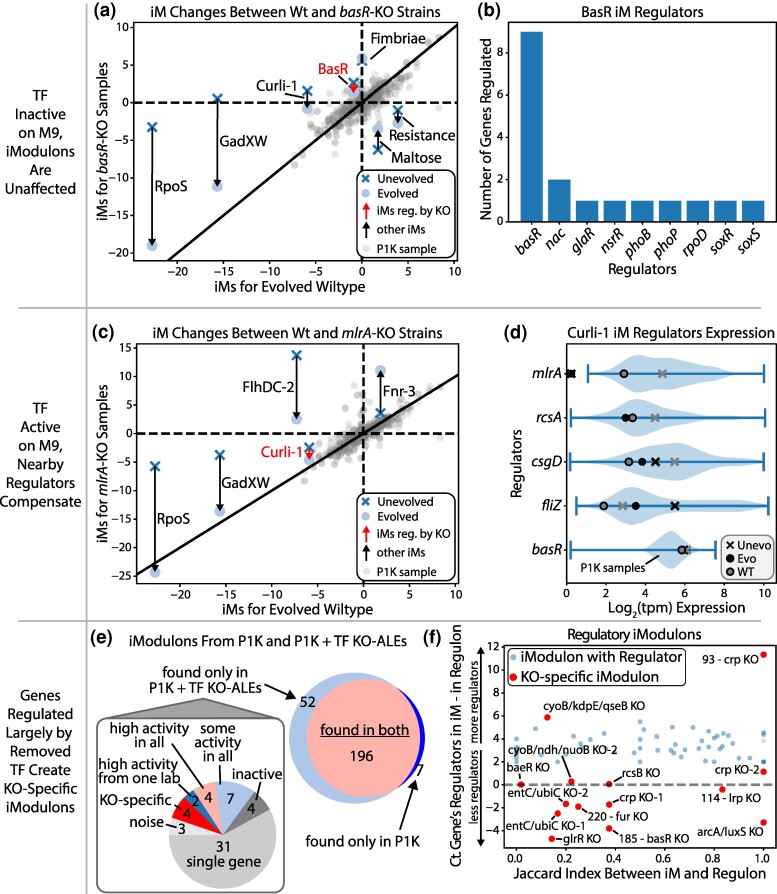
Mechanisms for recovery without regulator-specific mutations. a) Relative iModulon changes for all measured basR KO samples. iModulons that are differentially expressed or regulated by basR are labeled. b) The number of genes regulated by each regulator of the BasR iModulon, showing basR is the primary regulator of the iModulon. c) Relative iModulon changes for all measured mlrA KO samples. iModulons that are differentially expressed or regulated by mlrA are labeled. d) Differential expression of the regulators of Curli-1. The violin plots are of all samples of PRECISE1K. e) The iModulon pipeline was rerun with P1K and the samples from this study. Most of the iModulons remained the same (correlation of 0.6 or greater). f) All iModulons with regulators are shown alongside the KO-specific iModulons of both PRECISE1K and this study's samples.

For the majority of regulator KOs, the expression changes in their evolved strains were not genes regulated by the KO and the regulator's regulon was not enriched for mutations (see supplementary fig. S3, Supplementary Material online). *BasR*, for example, is the primary regulator of its eponymous iModulon which is largely unchanged during both the knockout and subsequent evolution (Fig. 2a). The BasR iModulon contains few other regulators (Fig. 2b), but as *basR* is not normally active on M9 the *basR* KO has little effect on the iModulon's activity. Of minor interest is that *basS*, the sensor kinase for *basR*, is extremely highly expressed in the *basR* KO samples although we have no clear explanation as for what effect this would have on the transcriptome. The lack of clear KO-specific expression differences or convergent mutations in these inactive regulator KO-ALE samples infers they are using nongenetic mechanisms to recover from their initial growth defect.

The Curli-1 iModulon, which is regulated by *mlrA*, is active on M9 but the *mlrA* KO and subsequent evolution has little effect on its expression (Fig. 2c). This is possibly due to the fact that Curli-1 contains many other regulators, some of whom are differentially expressed in the *mlrA* KO samples (Fig. 2d). It appears that other nearby regulators can help adapt to the loss of a TF in order to maintain normal iModulon activity levels.

The iModulon pipeline (Sastry et al. 2024) was run using the P1K dataset with samples from this study added, which resulted in new iModulons (Fig. 2e). There is a KO-specific iModulon for all but one of the regulator KO-ALEs with expression profiles (named in supplementary File S2, Supplementary Material online as 185 for the *basR* KO iModulon, 93—*crp*, 220—*fur*, and 114—*lrp*) which is common in KO studies (see supplementary table S2, Supplementary Material online). The genes in KO-specific iModulons often have fewer regulators than genes in most iModulons (Fig. 2f), showing that the lack of other nearby regulators leaves these genes largely unregulated which ICA captures as KO-specific iModulons.

### ALE Restores Growth-important Gene Expression When Deleted Regulators Were Active With Large Regulons

*Crp* (cyclic AMP receptor protein) regulates a wide variety of genes mostly encoding enzymes involved in carbon metabolism and transport (Gosset et al. 2004) and has long been the subject of intense interest in microbiology. *Crp* has the largest number of directly regulated genes (Tierrafría et al. 2022), but through evolution its KO can be adjusted for. This is possibly due to the fact that *crp* has relatively few targets of which it is the only regulator and therefore other regulators with overlapping targets are able to maintain normal expression of most growth-important genes.

**Fig. 3.**
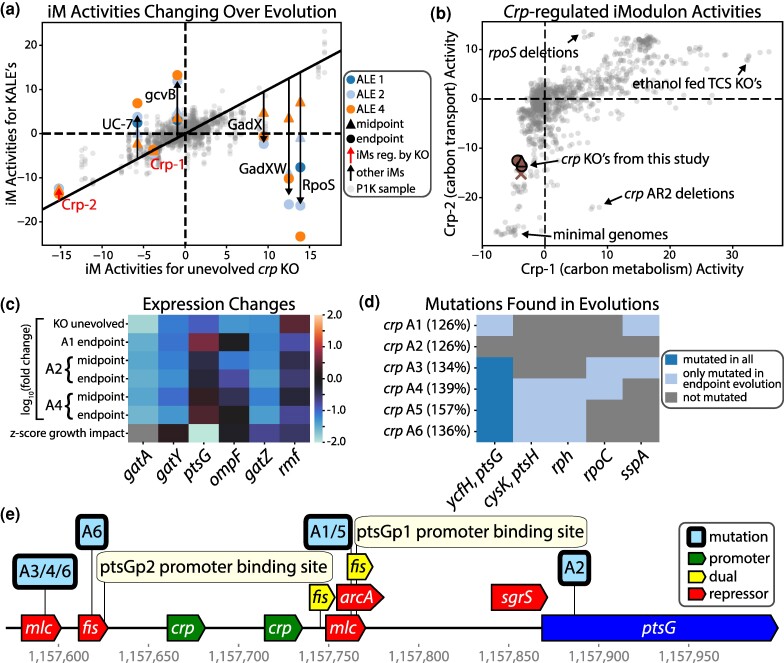
Laboratory evolution of the crp knockout strains resulted in targeted promoter mutations that restored normal expression of ptsG. a) Relative iModulon changes for all measured samples. iModulons that are differentially expressed are labeled. The relatively small amount of change to the crp iModulons over evolution shows that evolution does not restore the whole TRN but rather the genes with strong growth impacts. b) Comparison of Crp-1 and Crp-2 iModulon activities across PRECISE1K. Outliers are annotated. c) Only genes with differential expression in either the unevolved or evolved samples are shown. The most notable change is the restoration of ptsG to normal expression. All expression values are relative to the unevolved wildtype samples. The bottom row shows the impact to growth rate for the removal of each gene, which is available in supplementary File S3, Supplementary Material online. The expression profile of the midpoint sample for A1 is contaminated and is thus removed. d) All mutations that are found in at least two evolved strains are shown. There is a clear evolutionary selection pressure for ptsG promoter mutations. The percentages are the growth rate increase relative to the unevolved crp KO strain (i.e. crp A1 grows at a 2.36-fold higher rate). e) The promoter and repressor sites for ptsG and the location of the mutations for each of the sequenced endpoint strains.

Figure 3a shows the iModulon changes for both the *crp* KO and its evolutions. There are two *crp* iModulons (ignoring KO-specific iModulons) in P1 K whose activities are closely connected (Fig. 3b): Crp-1, which contains a large number of genes primarily involved in carbon metabolism and Crp-2, which is dominated by the *gatYZABCD* operon (a transporter of galactitol (Nobelmann and Lengeler 1996)). Crp-2 is substantially downregulated in both the unevolved and the evolved *crp* KO strains while Crp-1, which is inactive on M9 (see supplementary fig. S4, Supplementary Material online), is only minorly affected by the KO or evolution. This may be due to or enabled by the fact that Crp-1 and Crp-2 largely correspond to class I and class II *crp* binding, respectively (Lamoureux et al. 2023), thus showing that *crp* class II binding appears to be more active on minimal glucose media than *crp* class I binding.

The evolution does not restore normal expression of *crp*'s regulon on the whole (as measured by the Crp iModulons), but rather restores the expression of the most growth important genes such as *ptsG*. Figure 3c shows the most differentially expressed genes regulated by *crp* in the *crp* KO samples, which show evolution's ability to rebalance the most growth-important target of *crp* after its KO, *ptsG*. The *crp* evolution from our study shows similar results to a different *crp* KO-ALE study (Pal et al. 2022), both of which contain convergent mutations to *ptsG* repressor sites. The mutations found in the evolutions can be seen in Fig. 3d with convergent mutations upstream of *ptsG*, a vital gene for glucose import. These mutations, visualized in Fig. 3e, target repressor binding sites of other regulators.

*Crp* normally promotes *ptsG* and the KO thus severely downregulates the expression of *ptsG*, but the evolved samples are able to restore normal *ptsG* expression by reducing repressor activity. A2 is the only evolution without a *ptsG* mutation on a repressor site and instead has a mutation shortly downstream of the *ptsG* start codon. A2 is the slowest grower but this mutation does restore normal expression of *ptsG*, possibly through inhibiting *sgrS*, an sRNA which inhibits *ptsG* expression and binds near the start codon (Maki et al. 2010). These mutations are the first to arise in the strains, even before common M9 evolution adaptations such as RNAP mutations. This further implies there is a very strong selection pressure to restore normal *ptsG* expression within these evolutions.

**Fig. 4.**
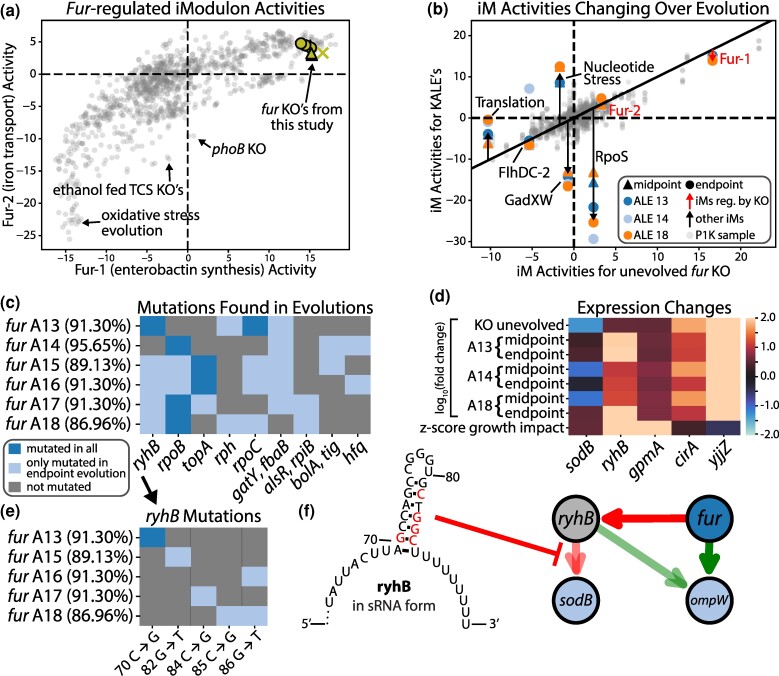
Laboratory evolution of the fur knockout strains resulted in targeted mutations to another regulator with a large TRN overlap. a) Comparison of Fur-1 and Fur-2 iModulon activities across PRECISE1K. Outliers are annotated. b) The differentially expressed iModulons for the evolved samples show that the majority of the evolution focuses on common growth-promoting adaptations rather than restoring the TRN to the normal state. c) Mutations found in at least two independent lineages are visualized and the percentages are the growth rate increases relative to the unevolved fur KO strain. d) Only genes with differential expression in either the unevolved or evolved samples are shown. All expression values are relative to the unevolved wildtype samples. The bottom row shows the impact to growth rate for the removal of each gene, which is available in supplementary File S3, Supplementary Material online. e) The specific sequence changes for the strains with an ryhB mutation are shown. f) The structural location of the ryhB mutations on the sRNA form of ryhB (Geissmann and Touati 2004), with red letters representing each mutated position. These mutations have been shown to reduce ryhB's ability to regulate its targets (Peterman et al. 2014).

*Fur* primarily acts as a regulator of iron transport/utilization and has a large regulon of 132 genes (Tierrafría et al. 2022), but the majority of the genes it regulates are also regulated by other TFs. There are two *fur* iModulons in P1 K whose activities are highly coordinated with each other (Fig. 4a): Fur-1 which is dominated by the *entCEBAH* operon which helps synthesize enterobactin to provide iron for metabolic pathways (Liu, Duncan, and Walsh 1989) and Fur-2 which consists of ABC iron transport proteins. Similar to the *crp* evolutions, the *fur* KO and evolution drastically changes the activity of Fur-1 while leaving Fur-2, which is less active on M9 (see supplementary fig. S4, Supplementary Material online), relatively unchanged. Instead of restoring normal expression of the *fur* iModulons (Fig. 4b), the evolved strains restore normal expression of high growth-impact genes, most notably *sodB* through mutations to *ryhB*.

*RyhB* is a sRNA which regulates many of the same targets that *fur* does and is normally repressed by *fur* (Seo et al. 2014). In the unevolved *fur* KO, *ryhB* is unrepressed and thus highly expressed which in turn severely represses *sodB*, a superoxide dismutase (Fig. 4d). The evolution has convergent mutations to some common ALE targets, such as RNAP and *topA* (Fig. 4c), but also mutates a specific region of *ryhB* (Fig. 4e). This region of *ryhB* is known to play an important role in *sodB* regulation and changes to it have been shown to reduce its ability to repress *sodB* (Peterman, Lavi-Itzkovitz, and Levine 2014). A13 is the only lineage with such a mutation in its midpoint evolution, which is shown to be the only midpoint evolution with normal expression of *sodB*. Interestingly A14 recovered growth with only a 5.3% representation of this mutation in its endpoint evolution population sample, showing there are possibly multiple potential adaptation strategies to these KOs.

While these *ryhB* mutations are convergent among the independent lineages, unlike the *ptsG* mutations found in the *crp* KO samples, they are not commonly found in the midpoint samples and instead RNAP mutations are more often the first to become dominant. This further shows the high potential growth impact of RNAP mutations, which has been well documented (Choudhury et al. 2023).

### *Lrp* KO-ALE Does not Recover Growth and is Associated With a Large Interconnected Regulatory Network

*Lrp* (leucine-responsive regulatory protein) is a global TF which coordinates cellular metabolism functions with the nutritional state of the cell (Hung et al. 2002). *Lrp* notably regulates amino acid anabolism and catabolism (Brinkman et al. 2003), stationary phase adaptations (Tani et al. 2002), and nutrient transport (Ernsting et al. 1992) among many other metabolic processes.

**Fig. 5.**
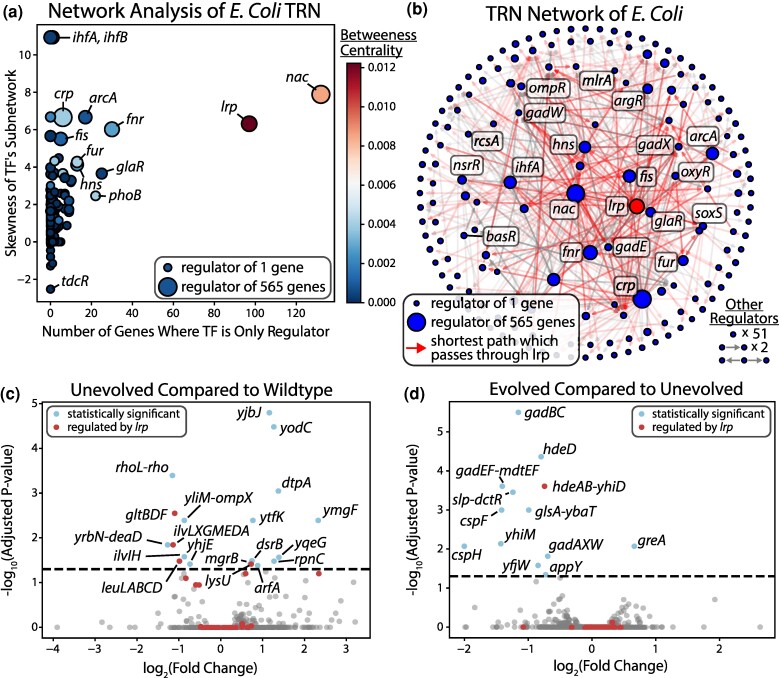
Laboratory evolution of the lrp knockout strains did not recover expression of its regulon. a) Skewness indicates the skew of the distribution of the number of degrees for each TF's regulon network, higher skew meaning more complexity. Betweenness centrality is a measure of how many other shortest paths between the regulators pass through a certain regulator. Lrp along with nac are outliers in all of these measures. b) The TRN network of *E. coli* according to RegulonDB (Tierrafría et al. 2022). Only genes that act as regulators are shown and regulatory networks unconnected to this central one are summarized in the bottom right (25.6% of regulators). While many genes are highly connected in the network visualized, lrp is a central element. All shortest paths between any two regulators that pass through lrp are colored red (60.4% of total shortest paths). c) A DEO (differentially expressed operons) plot comparing the unevolved lrp KO to the wildtype, showing the large effect of the KO. d) A DEO plot comparing the fastest endpoint strain (A23) of the lrp KO-ALE to the unevolved lrp KO strain. The evolution is not able to modify the expression of the large majority of lrp's regulon.

Figure 5a shows a variety of measures about the TRN which make it clear that *lrp* is an outlier in many ways. It has the second largest set of genes for which it is the only known regulator, its network is highly complex (inferred by the high skewness of its subnetwork), and its high betweenness centrality shows how a majority of the pathways between regulators pass through it. Figure 5b shows a directed network of regulatorily interconnected TFs which includes 74.4% of all regulators. In this network, 28.9% of all TF-TF pairs are connected through regulation. If *lrp* is removed from this network, this percentage drops by 54%. For reference, if *nac* is removed this drops by 41.2%, *crp* drops by 22.5%, and *fur* drops to 12.1%. *Lrp* is in a unique position in the TRN as one of if not the only regulator that if removed severely disrupts the network structure of TF–TF regulation.

The *lrp* KO-ALE strains stand alone among our KOs as being unable to recover growth. There are no TF-specific convergent mutations among its evolved lineages and what mutations do exist are limited to well-studied growth-promoting ones such as RNAP mutations (Dalldorf et al. 2023) (see supplementary fig. S5, Supplementary Material online). Transcriptional changes are largely limited to a downregulation of stress-related genes and slight upregulation of ribosomal subunits, which are common across ALEs (Dalldorf et al. 2023). The growth rate does improve through evolution, although this evolution contains no adaptations specific to the KO itself.

*Lrp* regulates numerous operons, many of whose expression is largely changed through *lrp*'s KO but unchanged by the subsequent evolution. Some of these operons are highly growth important, such as *gltBDF* and *livKHMGF* which the KO severely downregulated (Fig. 5c). The evolution was unable to rebalance these operons to return the transcriptome to a healthy state (Fig. 5d), thus limiting its growth capabilities.

### Regulator KOs and Subsequent Evolutions Affect Readiness of Regulator-associated Substrates

**Fig. 6.**
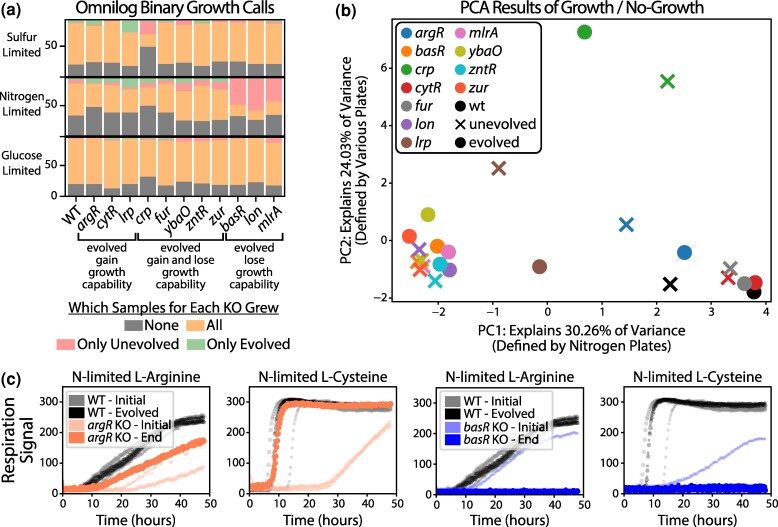
Non-M9 changes are present among the unevolved and evolved strains. a) The substrate readiness results for all of the KO-ALEs, showing that evolution overall reduces the ability of a strain to grow on new substrates as it focuses on increasing growth for the substrate it evolved on. See Methods: Biolog Plates and Analysis for more information. b) A principal components analysis carried out on the binary growth/no-growth calls of the phenotyping plates results. The top two highest variance-explaining principal components are shown. c) A selection of the growth profiles from the substrate readiness plates, showing the growth capability differences between the argR and basR KO strains on nitrogen-limited amino acid plates.

Despite the lack of convergent mutations or KO-specific transcriptional changes among many of the KO-ALE strains, the substrate readiness plates show distinct phenotypic changes between the wildtype, unevolved KO, and evolved KO strains. The plates showed that most of the evolved strains grew on fewer substrates than their unevolved ancestors as they specialized towards growth on minimal media supplemented with glucose when compared to the wildtype samples from this study (Fig. 6a). The strains that largely gain growth capabilities (*argR, cytR,* and *lrp* KOs) do not have regulator-specific mutations. Many strains show a reduction in ability to grow in nitrogen-limited conditions over the course of their evolution (*lon* KO evolution loses the ability to grow on 74.63% of nitrogen-limited conditions, *basR* 69.84%, *mlrA* 63.93%, *zur* 8.06%, *zntR* 7.46%, *ybaO* 4.92%, WT 3.45%), presumably a consequence of their evolution on nitrogen rich M9 media. It should be noted, however, that these growth losses seen largely in the *basR, lon,* and *mlrA* KO strains may also be experimental artifacts due to the lower oxygenation of our study's growth conditions when compared to the plate-based growth in Campos et al. (2018).

A principal component analysis of the binarized phenotype data indicates that the highest variance amongst the strains is explained by differences in nitrogen substrate utilization (Fig. 6b). The second highest variance explanatory component represents variance in carbon, nitrogen, and sulfur utilization. The *argR, crp,* and *lrp* KO-ALE strains exhibit the largest shifts in substrate utilization (see supplementary table S3, Supplementary Material online). While the changes for *crp* and *lrp* KO-ALEs were previously discussed, this large shift in substrate utilization is unexpected as the *argR* KO strains showed no convergent mutations and quickly recovered growth.

The unevolved and evolved KO strains for *argR*, a dual-regulator of arginine import and biosynthesis (Caldara et al. 2006), modify its growth capability on numerous amino acids. The unevolved *argR* KO strain loses the ability to grow on five amino acids under nitrogen-limited conditions that the wildtype can grow on (arginine, asparagine, cysteine, glutamic acid, and lysine). The evolved *argR* KO strain restores growth on four of these including arginine and increases growth rates on many of the other amino acids (see supplementary fig. S6, Supplementary Material online) (Caldara et al. 2006). Contrasting with the *argR* case, the fastest *basR* KO-ALE endpoint strain does not gain the ability to grow on any new substrates and instead loses the ability to grow on nearly any amino acid (supplementary fig. S7, Supplementary Material online). A few of these nitrogen-limited amino acid cases can be seen in Fig. 6c.

Some strains such as the *cytR* KO show few differences between the unevolved and evolved strains. The *cytR* KO strains show no change compared to the wildtype samples on any of the 6 cytidine conditions, which modulates *cytR* activity (Perini et al. 1996). *YbaO* (*decR*), which is thought to play a role in cysteine detoxification (Shimada et al. 2016), however showed distinct differences on nitrogen-limited cysteine conditions but relatively little difference caused by the evolution. Similar to what we found for some of the regulators that are active on minimal glucose media, it appears that activity alone is not enough to determine if a KO will lead to a detrimental effect on growth.

## Discussion

TFs have a complex evolutionary history whose regulatory targets vary greatly from species to species and regulator to regulator (Price et al. 2007). Regulator KO-ALEs reveal a wide variety of transcriptional, sequence, and phenotypic adaptations which are highly dependent on the activity of the regulator on the growth medium and the size of the regulator's regulon. Most of the regulator KO strains were able to recover growth rates through non regulator-specific evolutionary strategies. Some of these general recovery strains, despite an initial growth defect, are not active on minimal glucose media and the cell was able to restore growth without large changes to its TRN. Others, such as the *mlrA* KO-ALE strains, recovered growth through the utilization of regulators with overlapping targets to the removed regulator without the requirement of regulator-related mutations. The *crp* and *fur* KO evolved strains recovered growth both through common minimal media adaptations and by restoring the expression of highly growth-important genes through convergent mutations to elements of their own TRNs, while leaving the majority of their regulons highly differentially expressed. The *lrp* KO strains stand alone as being unable to recover growth, which is likely a consequence of *lrp*'s unique central position within the TRN.

*Escherichia coli* can restore growth through evolution without KO-specific mutations for most of the regulators included in this study. Often this is because the regulator is not normally active on M9, but the highly connected nature of the TRN means that even for many active regulator KOs, their regulons are still regulated by other TFs. The changes in expression or sequence through the evolution of these regulator KOs are not related to the removed regulator's regulon and instead represent evolutionary adjustments to M9 supplemented with glucose seen across a wide variety of ALEs (Dalldorf et al. 2023). An evolved 24% reduced genome derivative of MG1655 grew only 13% slower than evolved wildtype whereas the unevolved reduced genome strain grew 69% slower (Choe et al. 2019), inferring that the unexplained initial growth defect seen in many of our study's strains may be more general KO-response rather than a regulator KO-specific response since few regulator-related mutations or expression changes were observed for the majority of knockouts in this study.

Some regulator KO-ALEs, such as *crp* and *fur*, are able to recover growth through convergent mutations. *Crp* KO-ALE strains restore normal expression of *ptsG* through targeted mutations to repressor binding sites upstream of *ptsG* (in agreement with previous studies (Lamrabet et al. 2019; Pal et al. 2022)) and *fur* KO-ALE strains mutate a specific region of *ryhB* which helps rebalance *sodB* expression to standard levels (Peterman et al. 2014). Both the *crp* and *fur* KO-ALE strains, however, do not return their respective regulons to normal levels but rather restore the expression of the few most growth-important genes. A *pdhR* KO-ALE on glucose minimal media resulted in convergent mutations to elements of its TRN (Anand et al. 2021) similar to the *crp* and *fur* KO-ALEs from this study. *PdhR* has a relatively small regulon of 53 genes but contained in these are highly growth-important genes such as the pyruvate dehydrogenase complex (Anand et al. 2021). *PdhR* has no clear iModulon in order to infer its activity on said media but presumably is active and could thus be categorized alongside the *crp* and *fur* KOs.

*Lrp* KO-ALE is not able to restore normal growth, despite it being an evolutionary target in previous studies (Zinser and Kolter 2000; Ratib et al. 2021). This may be due to a handful of unique attributes of *lrp*, but most notably its large regulon of approximately one third of the genome (Kroner et al. 2019), the large number of genes of which it is the only regulator, and its central location within the TRN. Evolution is able to improve the growth rate of the *lrp* KO, but primarily through generic M9 growth-promoting mutations and expression changes that are seen across many samples from this study and numerous other ALE studies (Dalldorf et al. 2023). In a whole cell network study connecting together the TRN and metabolic networks, the Lrp-leucine complex was one of the central connections between the two networks, thus showing its central importance to not just regulating TFs but also its influence over metabolism (Grimbs et al. 2019).

Our characterization of substrate readiness showed a decreased ability of the evolved strains to grow on non-glucose substrates, so there are likely other conditions where these KOs may necessitate KO-specific adaptations. A study about novel ppGpp function found unexpected substrate readiness differences following gene knockouts (Gupta et al. 2015), giving some evidence that our observed growth differences seemingly unrelated to the removed regulator may actually be the result of said regulator's removal and not an experimental artifact. A large-scale study of *E. coli* phenotyping plates found the most no-growth calls on carbon sources and large disagreement of growth/no-growth calls on nitrogen sources (Mackie et al. 2014). That said, despite a lack of clear evolutionary adjustments, the evolution of regulator KOs did largely modify which conditions the strains could grow on, sometimes changing growth behavior on substrates related to the removed regulator.

Regulator KO-ALEs suggest a similar lesson to the metabolic KO-ALEs in that both create bottlenecks, in the TRN and in the metabolic map respectively, that are overcome using nearby existing connections and genetic mutations within these networks. We observed that relatively few mutations were necessary to recover growth when compared to other ALEs, suggesting a simplicity to genetic adaptations that enable phenotypic adjustment to the loss of a regulator (Phaneuf et al. 2020). An *nac* KO-ALE serves as a potential follow-up study to this one, as it, like *lrp*, regulates a large number of genes many of which have only it as a regulator and connects many TFs to each other in the TRN. Similar to how some of our removed TRN connections enabled new growth capabilities, another study showed how creating new regulatory links can also confer a growth benefit (Isalan et al. 2008). Additionally, the modification as opposed to removal of central regulators has also been shown to modify phenotype (Koubkova-Yu et al. 2018). Despite large initial growth impacts, it appears that the majority of regulators can be removed from *E. coli* on M9 minimal media and growth will quickly recover as the high connectivity of the TRN leaves it with few vulnerabilities. These results reinforce the long-standing view of the TRN as a highly adaptable network and begin to systematically uncover mechanisms by which this adaptability is achieved.

## Materials and Methods

### Strain Information

All strains were selected from the Keio collection (Baba et al. 2006). Round 1 strains (*argR*, *crp*, *cytR*, *fur*, and *lrp*) were evolved at the Center for Biosustainability at Denmark Technical University while round 2 strains (wt, *basR*, *lon*, *mlrA*, *ybaO*, *zntR*, and *zur*) were evolved at University of California San Diego.

### ALE and Growth Characterization

ALE was performed using 6 independent replicates of each regulator KO and the wildtype. All ALE experiments were conducted at 37 °C with a stirring speed of 1100 rpm for proper aeration. Each individual experiment was automatically passed to a new culturing flask at an OD_600_ nm = 0.6 using a passage volume of 100 μL. ALE was performed until no additional growth rate increase was observed, typically around 400 generations. Cultures were always maintained in excess nutrient conditions assessed by nontapering exponential growth. The evolution was performed for a sufficient time interval to allow the cells to reach their fitness plateau. The growth medium for all samples was M9 minimal medium with 4 g/L glucose. The M9 minimal medium contained 1× M9 salts, 2 mM MgSO_4_, 100 μM CaCl_2_, and 1× trace elements and Wolfe's vitamin solution. Stock M9 salts solution consisted of 10 × 68 g/L Na_2_HPO_4_ anhydrous, 30 g/L KH_2_PO_4_, 5 g/L NaCl, and 10 g/L NH_4_Cl dissolved in Milli-Q filtered water. M9 trace elements stock was a 2000× solution with composition of 3.0 g/L FeSO_4_·7H_2_O, 4.5 g/L ZnSO_4_·7H_2_O, 0.3 g/L CoCl_2_·6H_2_O, 0.4 g/L Na_2_MoO_4_·2H_2_O, 4.5 g/L CaCl_2_·H_2_O, 0.2 g/L CuSO_4_·2H_2_O, 1.0 g/L H_3_BO_3_, 15 g/L disodium ethylene-diamine-tetra-acetate, 0.1 g/L KI, 0.7 g/L MnCl_2_·4H_2_O, and concentrated HCl dissolved in Milli-Q filtered water.

Samples are named in the following convention: regulator KO’d, A(LE) number, F(lask) number, I(solate) number, R(eplicate) number. For example, argR A1 F14 I1 R1 is the A1 independent lineage of the *argR* knockout strain and is the first replicate from the first isolate taken from the 14th flask. Later flask numbers indicate longer evolution times. All knockouts also have an A0 strain, which is the unevolved sample. Throughout the article, endpoint flask refers to the higher flask number from a lineage while the midpoint flask refers to the other non-zero flask number from the lineage.

### DNA-sequencing

A clone from the midpoint and endpoints of the evolved strains was picked for DNA sequencing. Population samples were also sequenced for endpoint evolved strains. The sequence mutations found in the population and endpoint isolates have a high overlap, with an average Jaccard index 0.6042. The strains were grown in an M9 minimal medium supplemented with 4 g/L glucose. Total DNA was sampled from an overnight grown culture at an OD_600_ nm = 0.6. Nucleic acid isolation, library preparation, and subsequent analysis were performed as previously described (Anand et al. 2019). Briefly, genomic DNA was isolated using a Nucleospin Tissue kit including treatment with RNase A. Resequencing libraries were prepared following the manufacturer's protocol using Nextera XT kit. Sequencing was performed on an Illumina HiSeq. Sequence data is available at https://aledb.org/ale/project/138/. A target read depth of 100 × was obtained for each sample. Clonal isolates were sequenced for midpoint and endpoint strains. Additionally, population sequencing was performed for the endpoint strains. When available, mutations used in this study are pulled from the population sequences.

### Mutation Calling

Sequencing reads were filtered and trimmed using AfterQC version 0.9.7 (Chen et al. 2017). We mapped reads to the *E. coli* K-12 MG1655 reference genome (NC_00913.3) using the breseq pipeline version 0.33.1(Deatherage and Barrick 2014). Consensus frequency cutoff was set to 0.75 and required match fraction set to 0.95. The breseq pipeline was run in clonal or population mode depending on the sample type. Mutation analysis was performed using ALEdb (Phaneuf et al. 2019). An average of 7.3 mutations were found in each isolate, when excluding the hypermutator strain (crp A3 F87), which converts to a mutation in 0.0001825% of all base pairs. On average we observed a coverage depth of 88 reads for sequenced strains. More details are available in supplementary File S4, Supplementary Material online.

### RNA-sequencing

All samples were prepared and collected in biological duplicates at UCSD, with two libraries prepared for each of the two replicates for each sample. Three milliliter of culture sampled from an overnight grown culture at an OD_600_ nm = 0.5 was added to 6 mL of Qiagen RNA-protect Bacteria Reagent after sample collection. This solution was then vortexed for 5 s, incubated at room temperature for 5 min, and then centrifuged. The supernatant was then removed and the cell pellet was stored at −80 °C. The Zymo Research Quick-RNA MicroPrep Kit was used to extract RNA from the cell pellets per vendor protocol. On-columns DNase treatment was performed for 30 min at room temperature. Anti-rRNA DNA Oligo mix and Hybridase Thermostable RNase H (Choe et al. 2021) was used to remove ribosomal RNA. Sequencing libraries were created using a Kapa Biosystems RNA HyperPrep per vendor protocol. RNA-sequencing reads were processed using https://github.com/avsastry/modulome-workflow. Data are available at NCBI GEO GSE266148.

### iModulon Computation

RNA-sequencing data were used to create iModulon activity levels of our strains using PyModulon (Sastry et al. 2019) which is available at https://github.com/SBRG/pymodulon. Activities of iModulons were compared to samples from PRECISE1K which is easily accessible using iModulonDB (Rychel et al. 2021). The calculations of new iModulons for our dataset in addition to PRECISE1K were performed using modulome-workflow (Sastry et al. 2024) which is available at https://github.com/avsastry/modulome-workflow. See https://imodulondb.org/ (Rychel et al. 2021) for a more complete description of iModulons and their calculation.

### Basal iModulon Transformation

Independent component analysis decomposes a matrix **X**, which is a collection of transcriptomic profiles ideally containing various perturbations of a particular species, into two matrices, **M** and **A**. The **M** matrix contains components called iModulons with dimensions of the number of genes; these iModulons are linked to specific regulators by overlap of their gene sets with experimentally-determined regulons. The **A** matrix contains the weights of the iModulons in the various samples. In the final generation of the **M** and **A** matrices within our workflow, the sign of specific components in M is typically inverted to ensure a predominantly positive distribution of gene weights. This adjustment involves reversing the sign of the corresponding columns in the **M** matrix and the associated rows in the **A** matrix. However, the interpretation of **A** matrix values remains uncertain as higher activity could mean either more or less regulation, depending on the weightings of genes in **M** and the biological role of the regulator. To improve the interpretability of the **A** matrix, we developed a “Basal iModulon transformation”, in which further adjustments were made such that higher values correspond to an increased regulatory activity (basal activity). Each iModulon was assigned a direction based on the function of its canonical regulator and, when available, the activity observed in knockout (KO) samples. To align the A matrix values with a baseline, the values were shifted such that the minimum approximates zero. This was achieved by subtracting the 95th quantile for iModulons with a positive direction, and the 5th quantile for those with a negative direction, ensuring minimal influence from outliers. If the direction was positive, the signs of both matrices A and M were subsequently flipped to maintain consistency.

### Biolog Plates and Analysis

The OmniLog system was used to generate the media screens. First overnight cultures were grown in 4 mL of M9 4 g/L glucose medium at 37 °C with shaking. Pellets were collected by centrifuge and pellets for PM01 plates were washed twice by M9 medium while pellets for PM03B and PM04A plates were washed twice using IF0a medium (supplied by OmniLog). 42%T and 85%T sample solutions were prepared in M9 no carbon medium with 1× DyeA (supplied by OmniLog) for PM01 plates. 42%T and 85% sample solutions were prepared in IF0a medium with 1× carbon (Na-succinate and FE-citrate) and 1× DyeA. One hundred microliters of 85% T sample solution were placed in each well. The plates were run on the OmniLog machine at 37 °C for 48 h. Opacity readings were generated of the plates over these 48 h.

In order to convert the opacity respiration readings to growth curves, the signal from every well is processed through a Savitzky-Golay filter to smoothen the data. A window length of 50 and polynomial degree of 3 is used for said filter. The maximum signal value is recorded and a control group is formed of the negative control wells. A one-sided z-test is performed to calculate *P*-values associated with each well and are corrected for multiple hypothesis tests using the Bonferroni correction. If the adjusted *P*-value is below 0.05 the well is considered to have a significant growth signal and is assigned to have grown, else no growth is assumed.

## Supplemental Material

Supplementary material is available at *Molecular Biology and Evolution* online.

## Supplementary Material

msae240_Supplementary_Data

## Data Availability

Additional Supplementary Data Files 1, 2, 3, and 4 (.csv and .xlsx) are publicly available online at https://zenodo.org/records/14052132. Raw data for ALE experiments are available at aledb.org.
