# Supplementary material for: Diversity of Transcriptional Regulatory Adaptation in E. coli
Source: Mol Biol Evol. 2024 Nov 12;41(11):msae240. doi: 10.1093/molbev/msae240 (PMC11588850; doi:10.1093/molbev/msae240)
Supplement: msae240_Supplementary_Data [file msae240_supplementary_data.pdf]

Supplementary Information

Supplemental Table 1: Initial list of targets for TF KO-ALE. *NikR* and *puuR* knockouts did not show a significant growth defect in our lab so were not fully evolved.

TF	Growth Defect from Keio[12] (%age of wildtype)	Size of Regulon[15]	Variance of TF's Regulon Explained by iModulons	Independent Lineages' Names (each has midpoint and endpoint isolate)	Average Number of Mutations per Endpoint Isolate	Samples Selected for RNA-seq (in addition to unevolved KO)	Sample Selected for Biolog Plates (in addition to unevolved KO)
argR	73.81%	37	90.69%	A1-A6	7.17	n/a	A5 endpoint
basR	89.75%	13	63.56%	A4-A9	6.34	A7 endpoint	A7 endpoint
crp	77.77%	576	83.20%	A1-A6	13.5* (w/o hyper-mutator 9.2)	A1 midpoint, A1 endpoint, A2 midpoint, A2 endpoint, A4 midpoint, A4 endpoint	A4 endpoint
cytR	83.06%	13	58.71%	A7-A12	5.67	n/a	A7 endpoint
fur	84.55%	132	89.76%	A13-A18	6.50	A13 midpoint, A13 endpoint, A14 midpoint, A14 endpoint, A18 midpoint, A18 endpoint	A13 endpoint
lon	79.37%	n/a	n/a	A16-A21	3.83	A17 endpoint	A17 endpoint
lrp	86.56%	71	90.42%	A19-A24	3.17	A23 endpoint	A23 endpoint
mlrA	89.83%	10	84.88%	A10-A15	2.34	A15 endpoint	A15 endpoint
nikR	92.93%	6	92.91%	n/a	n/a	n/a	n/a
puuR	92.31%	8	95.22%	n/a	n/a	n/a	n/a
wt (no KO)	n/a	n/a	n/a	A1-A6	1.34	A3 endpoint	A3 endpoint
ybaO	95.69%	n/a	n/a	A10-A15	3.83	n/a	A11 endpoint
zntR	91.84%	1	77.51%	A16-A21	2.33	n/a	A16 endpoint
zur	91.72%	5	94.44%	A4-A9	1.83	n/a	A7 endpoint

Supplemental Table 2: iModulons generated by specific knockouts along with what perturbation activates it and the source study of this perturbation. Low activity means highly negative activity values. iModulons are

relative and zero activity is equal to expression levels for strains growth on M9 glucose and isolated in the exponential growth phase.

**iModulon**	**Genetic Perbuation Which Activates It**	**Study Name on P1K Where Most Activated**
arcA/luxS KO	*arcA* KO gives low activity, luxS KO gives high activity	DDB2
baeR KO	*baeR* KO gives low activity	DDB3
crp KO-1	*crp* KO gives high activity, KOing one one or both of crp's activating regions leads to low activity	Crp ARs
crp KO-2	KOing *crp* AR2 leads to high activity, KOing *crp* leads to low activity	Crp ARs
cydB/appC KO	*cydB* and *appC* KO leads to low activity	EEP
cyoB/ndh/nuoB KO-1	*cyoB*, *ndh*, and *nuoB* KO leads to both high and low activity	EEP
cyoB/ndh/nuoB KO-2	*cyoB*, *ndh*, and *nuoB* KO leads to both high and low activity	EEP
cyoB/kdpE/qseB KO	*cyoB*, *kdpE*, and *qseB* KO leads to low activity	DDB2
entC/ubiC KO-1	*entC* and *ubiC* KO leads to low activity	EEP
entC/ubiC KO-2	*entC* and *ubiC* KO leads to low activity	EEP
glrR KO	*glrR* KO leads to low activity	DDB2
lsrB KO	*lsrB* KO leads to high activity	DDB3
nquinone ALE 3	*ubiC* KO evolution leads to low activity	nquinone
pgi KO	*pgi* KO leads to low activity	CCK_pgi, SvNS PGI
pts ALE	*pts* KO evolution leads to low activity	CCK_pts
pts KO	*pts* KO leads to low activity	CCK_pts
rcsB KO	*rcsB* KO leads to high activity	TCS
sdh KO	*sdh* KO leads to low activity	CCK_sdh
thrA KO	*thrA* KO leads to low activity	False Positives
tpiA KO	*tpiA* KO leads to high activity	CCK_tpiA

Supplemental Table 3: TF KO-ALE targets and shift between unevolved and evolved isolates growth on Biolog plates.

TF	Jaccard Index of Substrate Conditions that Unevolved and Evolved Isolates Grow
argR	0.8677
basR	0.9375
crp	0.6266
cytR	0.8990
fur	0.9117
lon	0.9375
lrp	0.7912
mlrA	0.8757
wt (no KO)	0.9375
ybaO	0.8679
zntR	0.9107
zur	0.9371

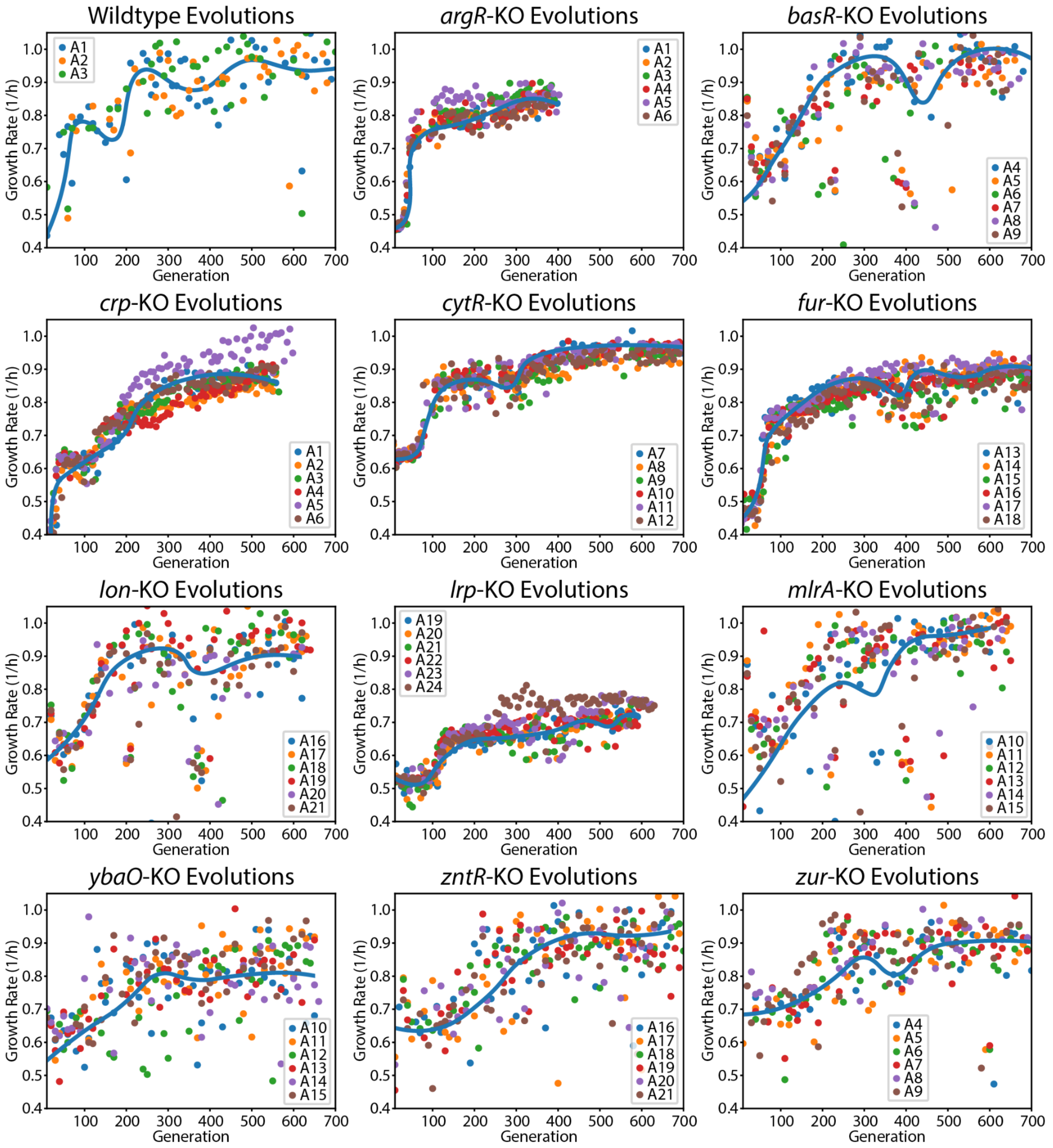

Supplemental Figure 1 - Growth rate plots of all independent lineages of the knockouts. The independent lineages of each of the 11 TF KO-ALE's and the wildtype samples are shown. Some experiments, such as *argR*, were stopped early due to growth rates approaching wildtype and a lack of growth change.

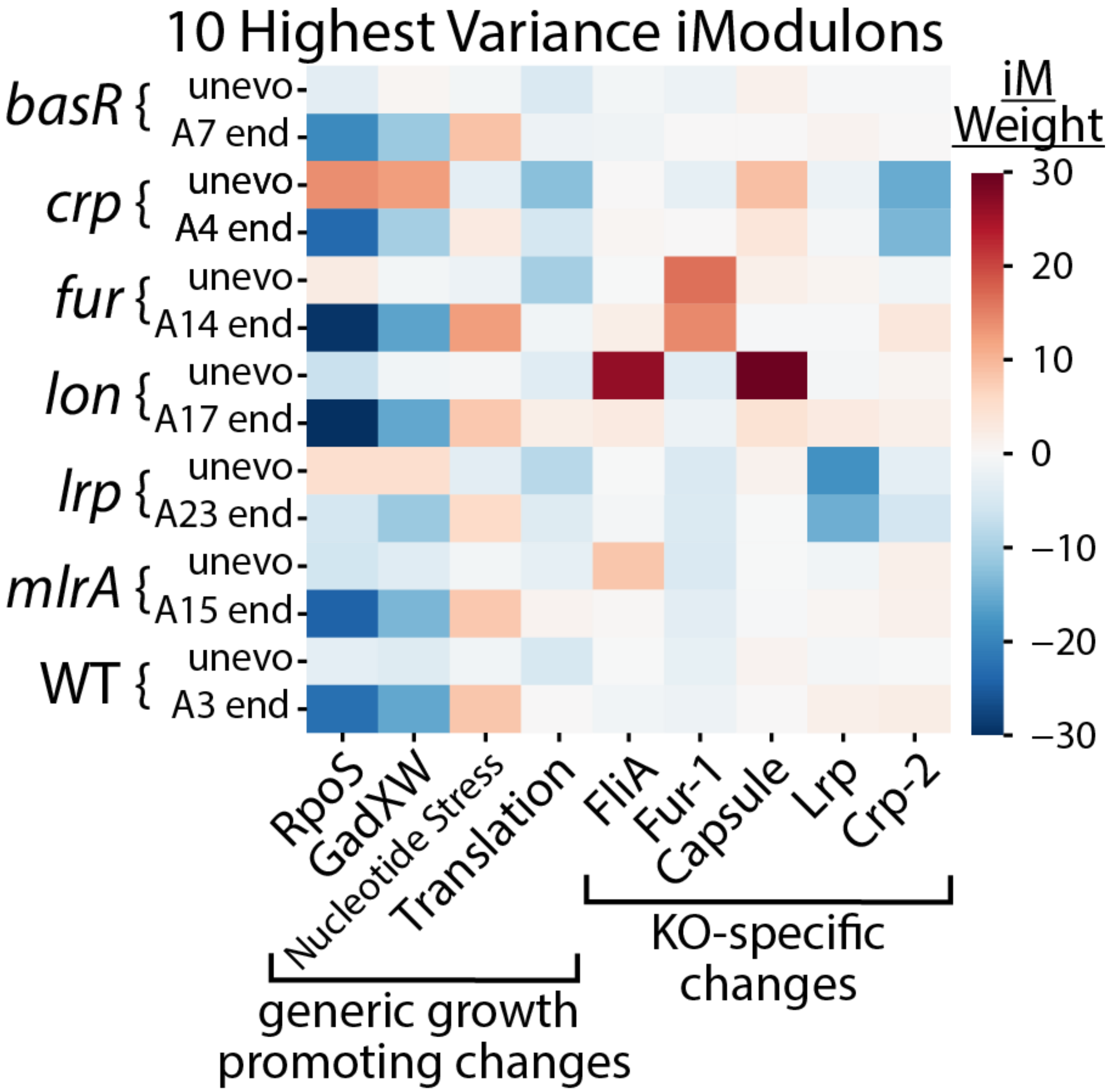

Supplemental Figure 2 - Highest variance iModulons among the expression profiled samples. The top nine iModulons with the highest variance activity among the samples from this study are shown, alongside the Translation iModulon.

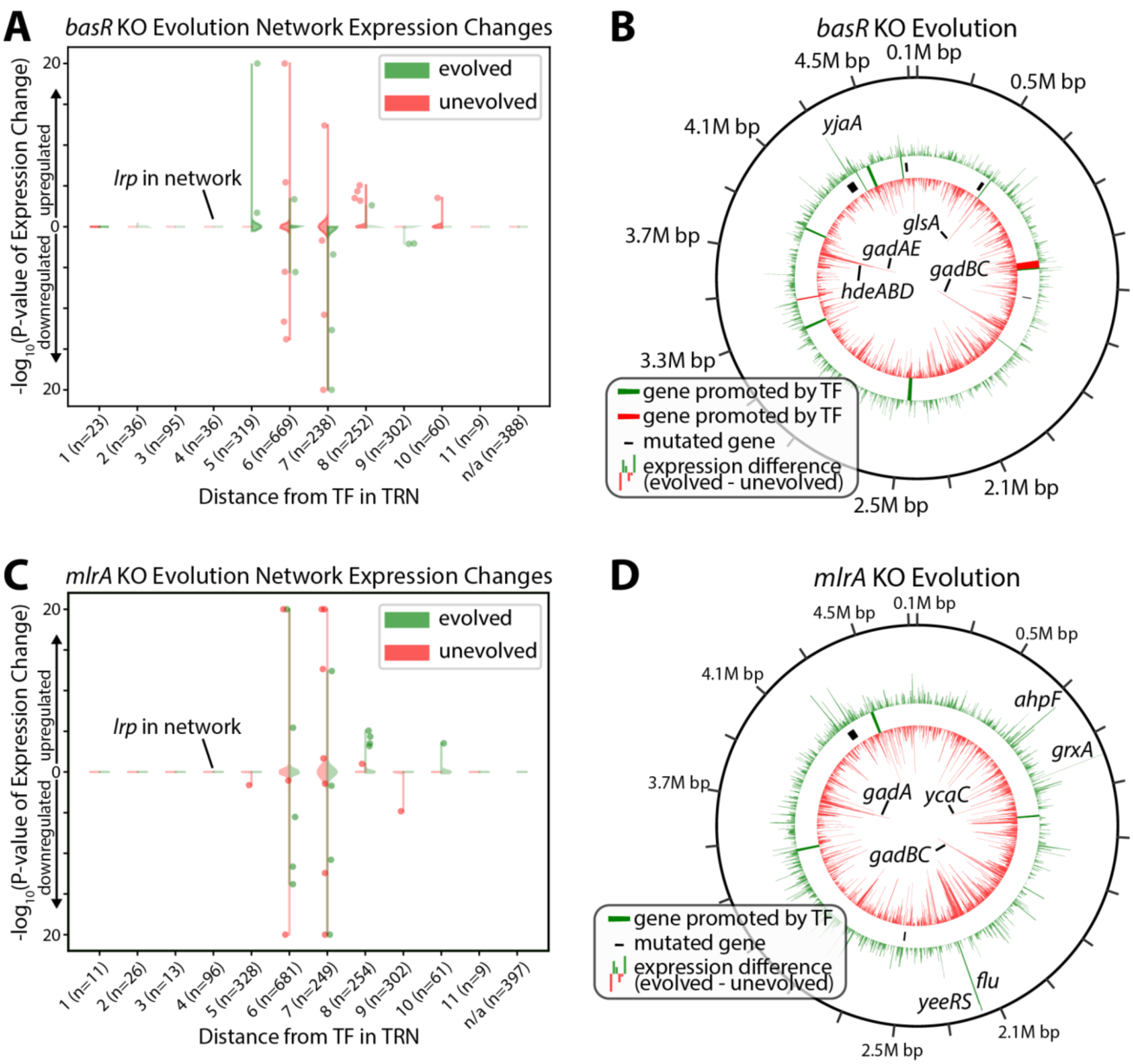

Supplemental Figure 3: (A) The vertical axis shows the expression change in genes when compared to the unevolved wildtype sample, with the red and green violin plots representing the unevolved and evolved *basR* KO-ALE respectively. Moving from left to right, the first column are all the directly regulated genes of *basR*, the second column are all regulatory targets of the first column's genes, and so on until no more genes can be reached leaving 388 genes that are not connected to *basR*. **(B)** The middle ring shows where in the chromosome mutations and *basR*'s regulon are while the red and green activities represent transcriptional changes for the *basR* KO-ALE. **(C)** Same as panel A, but constructed for *mlrA*. **(D)** Same as panel B, but constructed for *mlrA*.

crp, Crp-1

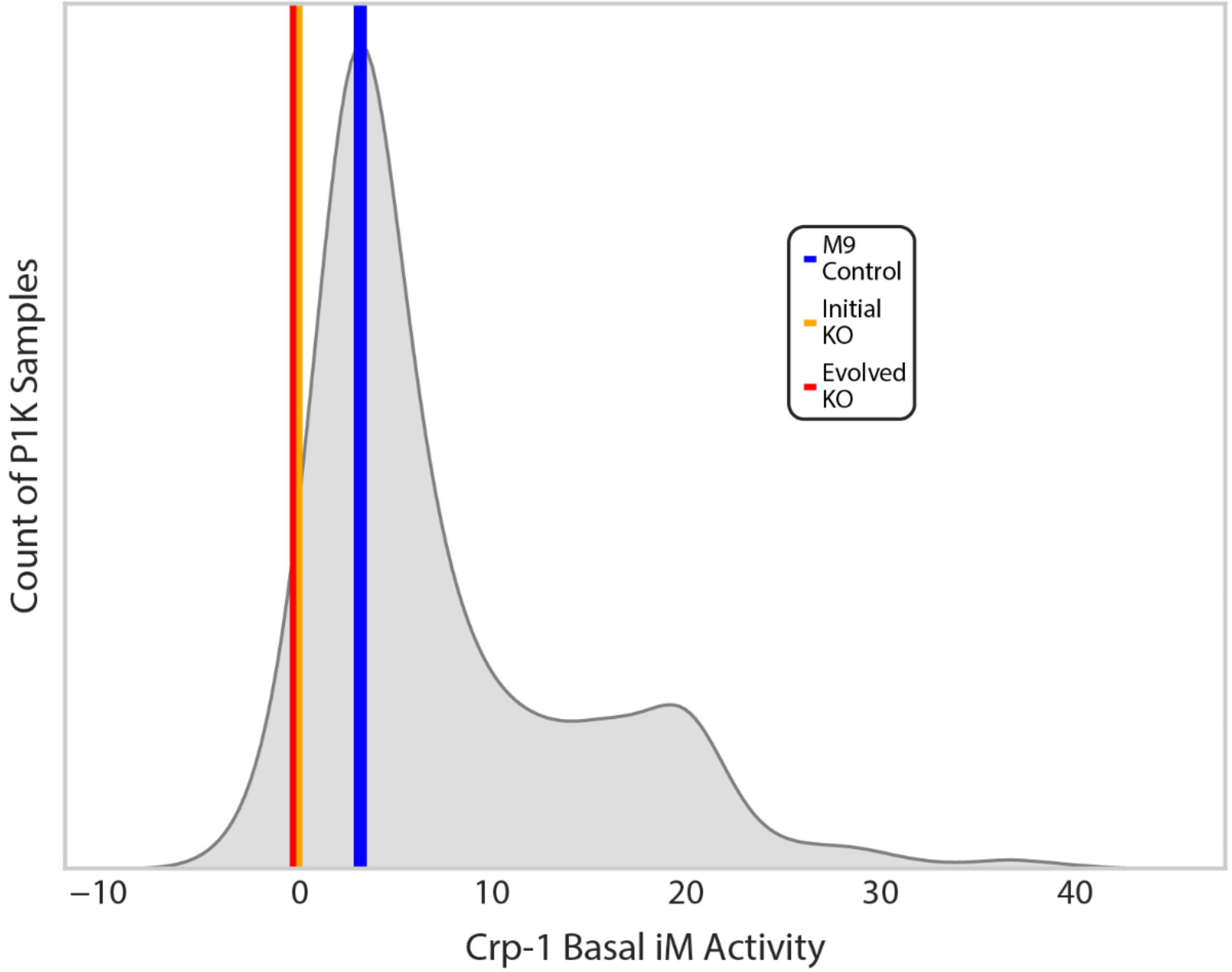

fur, Fur-1

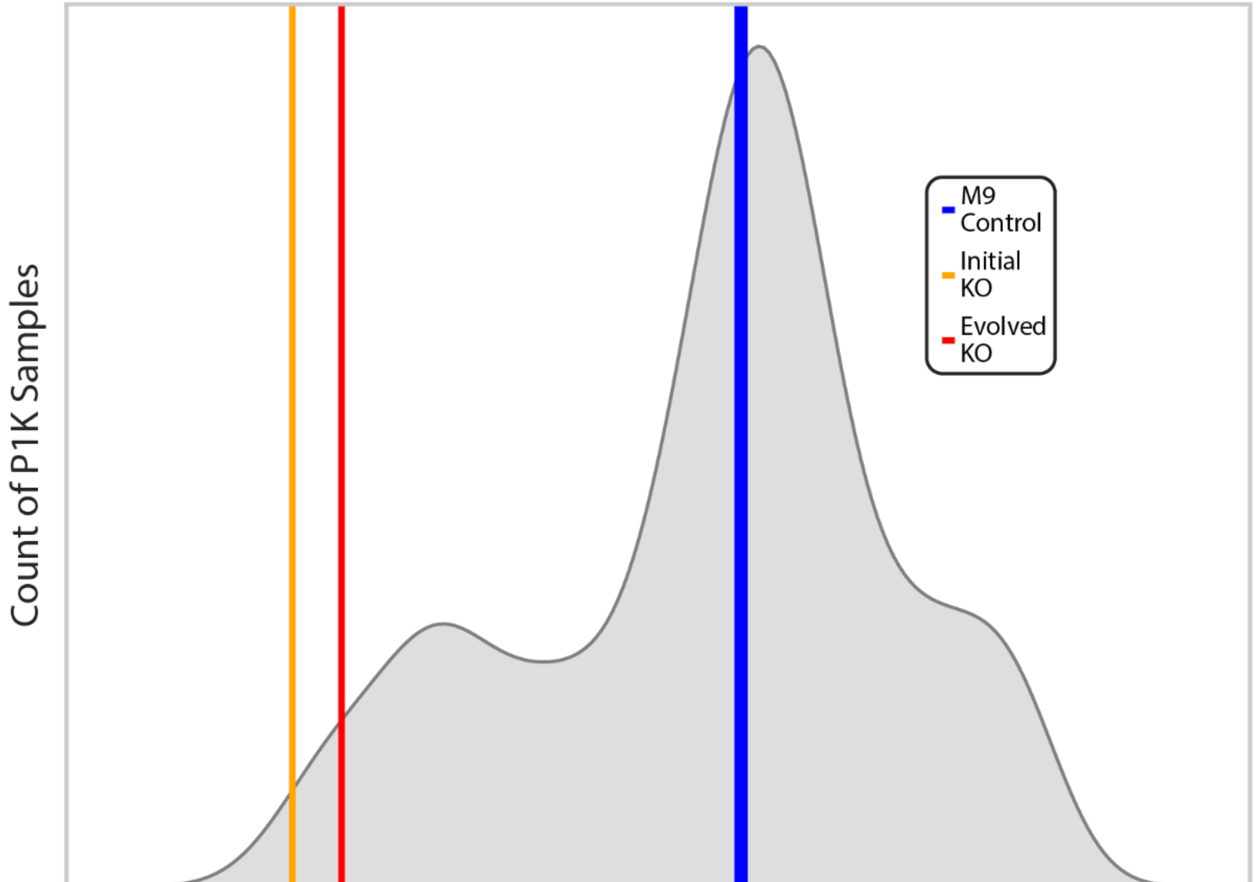

Supplemental Figure 4 - Crp-1 and Fur-1 basal-adjusted iM activity. Density plot of the Crp-1 and Fur-1 basal-adjusted iModulon's activity level across PRECISE1K samples.

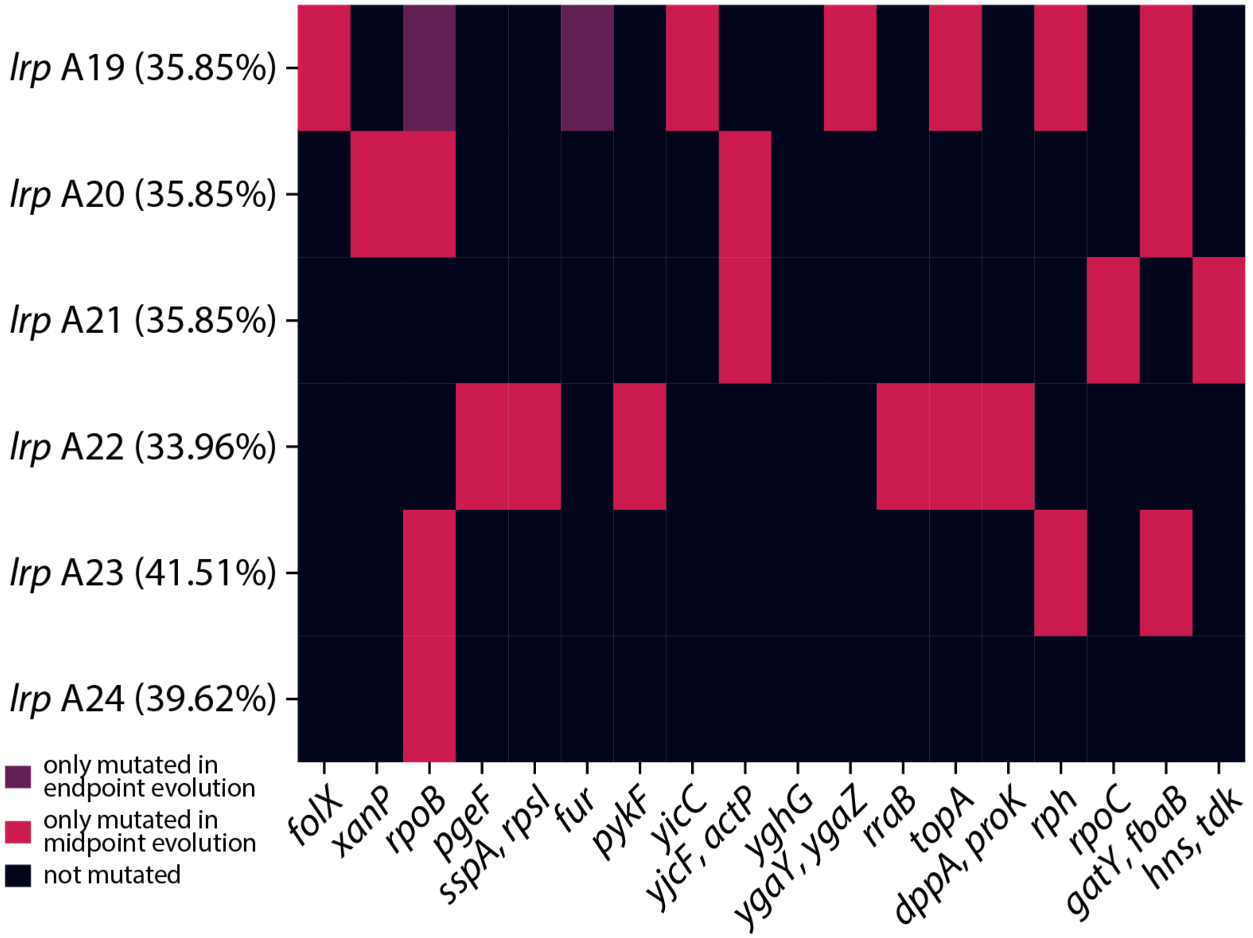

Supplemental Figure 5 - *Lrp* KO-ALE mutations: Mutations found in *lrp* KO-ALEs across all samples. Only mutations found in at least two samples are shown.

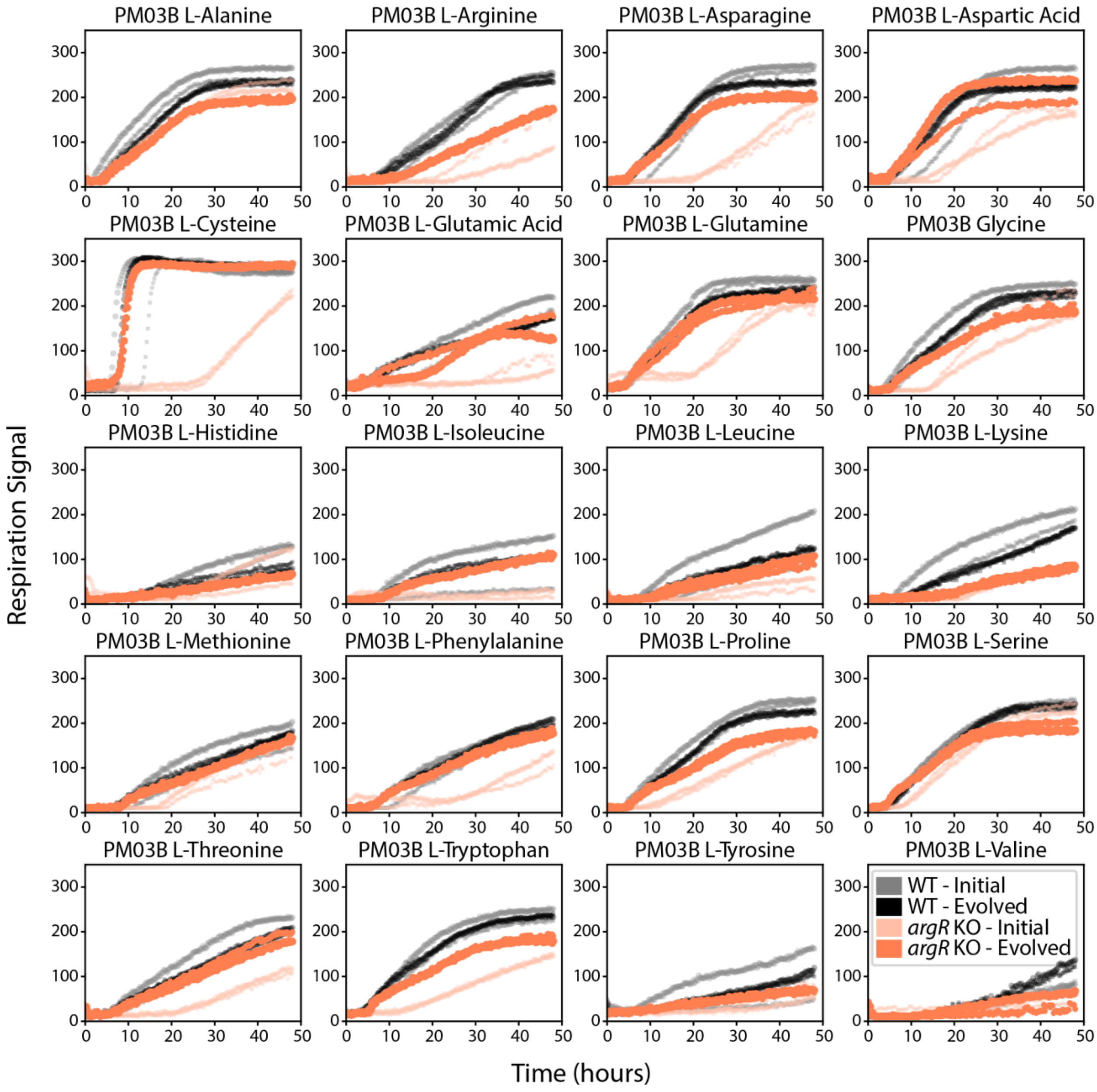

Supplemental Figure 6: Respiration signals is an opacity reading that is the measurement for growth for OmniLog plates. Each plot here represents a different nitrogen-limited plate supplemented with an amino acid.

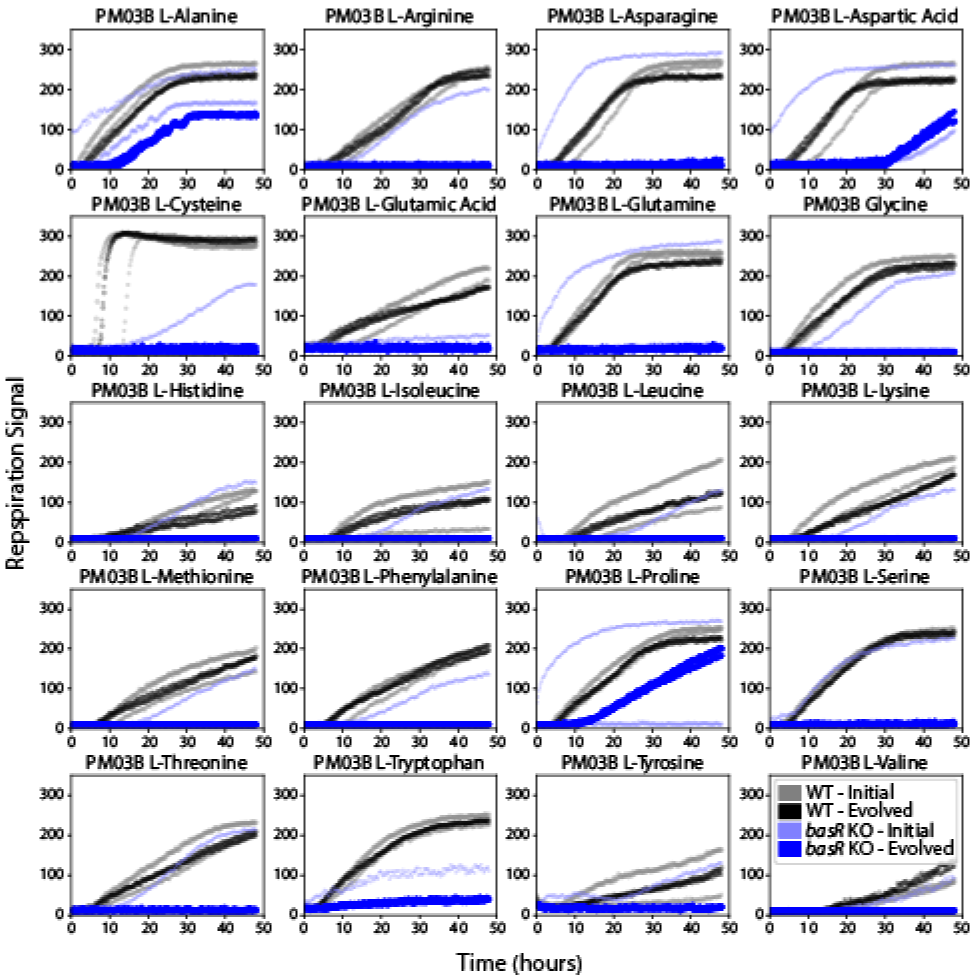

Supplemental Figure 7: Respiration signals is an opacity reading that is the measurement for growth for OmniLog plates. Each plot here represents a different nitrogen-limited plate supplemented with an amino acid.
